# *WRN* Germline Mutation Is the Likely Inherited Etiology of Various Cancer Types in One Iranian Family

**DOI:** 10.3389/fonc.2021.648649

**Published:** 2021-06-07

**Authors:** Mahnaz Norouzi, Mohammad Shafiei, Zeinab Abdollahi, Paniz Miar, Hamid Galehdari, Mohammad Hasan Emami, Mehrdad Zeinalian, Mohammad Amin Tabatabaiefar

**Affiliations:** ^1^ Department of Biology, Faculty of Sciences, Shahid Chamran University of Ahvaz, Ahvaz, Iran; ^2^ Department of Genetics and Molecular Biology, School of Medicine, Isfahan University of Medical Sciences, Isfahan, Iran; ^3^ Department of Gastroenterology, Poursina Hakim Digestive Disease Research Center, Isfahan University of Medical Sciences, Isfahan, Iran; ^4^ Pediatric Inherited Diseases Research Center, Research Institute for Primordial Prevention of Noncommunicable Disease, Isfahan University of Medical Sciences, Isfahan, Iran

**Keywords:** colorectal cancer, microsatellite instability, hereditary non-polyposis colorectal cancer, next-generation sequencing, *WRN*

## Abstract

**Background:**

Familial cancers comprise a considerable distribution of colorectal cancers (CRCs), of which only about 5% occurs through well-established hereditary syndromes. It has been demonstrated that deleterious variants at the newly identified cancer-predisposing genes could describe the etiology of undefined familial cancers.

**Methods:**

The present study aimed to identify the genetic etiology in a 32-year-old man with early onset familial CRC employing several molecular diagnostic techniques. DNA was extracted from tumoral and normal formalin-fixed-paraffin-embedded (FFPE) blocks, and microsatellite instability (MSI) was evaluated. Immunohistochemistry staining of MMR proteins was performed on tumoral FFPE blocks. Next-generation sequencing (NGS), multiplex ligation-dependent amplification (MLPA) assay, and Sanger sequencing were applied on the genomic DNA extracted from peripheral blood. Data analysis was performed using bioinformatics tools. Genetic variants interpretation was based on ACMG.

**Results:**

MSI analysis indicated MSI-H phenotype, and IHC staining proved no expressions of MSH2 and MSH6 proteins. MLPA and NGS data showed no pathogenic variants in MMR genes. Further analysis of NGS data revealed a candidate *WRN* frameshift variant (p.R389Efs*3), which was validated with Sanger sequencing. The variant was interpreted as pathogenic since it met the criteria based on the ACMG guideline including very strong (PVS1), strong (PS3), and moderate (PM2).

**Conclusion:**

*WRN* is a DNA helicase participating in DNA repair pathways to sustain genomic stability. *WRN* deficient function may contribute to CRC development that is valuable for further investigation as a candidate gene in hereditary cancer syndrome diagnosis.

## Introduction

Hereditary cancer syndromes account for a small percentage of all cancers diagnosed each year, although they are common in some cancer forms, such as colorectal and breast cancers (1, 2). Colorectal cancer (CRC) is considered one of the most frequent malignancies worldwide, and a family history of the condition is a major risk factor (1, 3). Notably, the relative risk of developing CRC increases in individuals with a family history of CRC. Alongside screening and surveillance, another advantage of determining the molecular basis of familial CRC is its therapeutic relevance (4, 5). Meanwhile, chromosomal instability arises in a remarkable portion of CRC tumors. Microsatellite instability (MSI), which is caused by the aberrant function of the DNA mismatch repair (MMR) system, accounts for nearly 15% of all CRC patients. In fact, insertion or deletion of a repetitive unit in a short tandemly repeated sequence (STR), designated as MSI, is a robust diagnostic marker for identifying Lynch syndrome (LS) suspicious individuals (6–8).

LS is an autosomal dominant hereditary cancer syndrome caused by germline defects in the DNA mismatch repair genes, including *MLH1*, *MSH2*, *MSH6*, *PMS2,* and *EPCAM* (9, 10). Most of the pathogenic variants of MMR genes include small insertions/deletions or large genetic rearrangements, missense, and nonsense variants (11–13). The majority of these gene defects were found in *MLH1* and *MSH2* (60 to 80%) genes. Other detrimental variants in *MSH6* or *PMS2* and, rarely, to *EpCAM* genes have been identified associated with Lynch phenotype. Nevertheless, recent population studies have found a higher frequency for germline pathogenic variants in *MSH6* and *PMS2* genes (14–16). It was demonstrated that these genes encompass lower penetrance pathogenic variants, and *MSH6* deleterious variant carriers represent late-onset cancer compared to *MLH1* or *MSH2 *carriers (13). Moreover, an increased risk of extracolonic tumors, particularly endometrial cancer, has been shown in *MSH2* pathogenic variant carriers (13). Endometrial and ovarian cancers are the most prevalent Lynch-associated tumors. Other associated cancers include pancreatic, biliary tract, ureter, renal pelvis, gastric, small intestine, brain, sebaceous adenomas, sebaceous carcinomas, and keratoacanthomas (9).

LS is also known as hereditary non-polyposis colorectal cancer (HNPCC), with an approximate prevalence of one in 600 to one in 3,000 individuals (17, 18). HNPCC is a heterogeneous group of disorders with overlapping features that are clinically evaluated using Amsterdam (I or II) or Bethesda guidelines (19–22). Clinical guidelines are based on family and personal history of cancer, and their use as diagnostic tools for LS is restricted due to their low sensitivity. Patients with HNPPCC are at increased risk for metachronous or synchronous colon and extracolonic Lynch-related cancers. Since many patients with Lynch syndrome (UP TO 50%) do not even meet the revised Bethesda guidelines, an alternative approach referred to as “universal tumor screening” is recommended. Both MMR immunohistochemistry (IHC) staining and MSI analyses (either by themselves or in combination) are used to test MMR system proteins in all newly diagnosed CRC patients (9, 10). MSI is not exclusive to the Lynch-related CRCs. It may be observed in other CRC tumors. Somatic *MLH1* gene inactivation due to promoter hypermethylation characterizes sporadic MMR deficient (dMMR) colorectal tumors, which typically arise in patients with late-onset cancer (12, 13). Unexplained dMMR CRC, termed “Lynch-like syndrome”, is another MSI colorectal tumor for which neither the deleterious germline variant of MMR genes nor the hypermethylation of the *MLH1* gene promoter can be identified (12, 15, 23). Somatic bi-allelic mutations in the MMR genes may explain a large number of these Lynch-like dMMR tumors (nearly 70%). However, the etiology of a large number of these unclassified MSI/dMMR tumors is unclear (12, 23, 24). Moreover, dMMR CRCs encompass around 60% of HNPCC cases. About 40% of HNPCC patients do not represent MMR defects, and they are accordingly introduced as familial colorectal cancer type X (FCCTX) with undefined etiology. FCCTX is a group of heterogeneous diseases characterized by microsatellite stable (MSS) and late-onset clinically different from LS (21, 25, 26). Despite the recent advances in gene identification technologies, the genetic etiology of many hereditary cancers remains undefined, especially FCCTX groups (27, 28).

By the poor clinical diagnostic approaches, it is difficult to differentiate Lynch CRC tumors from non-hereditary ones due to the high frequency of MSI occurrence in sporadic tumors. This situation becomes more complicated when there is a positive family history of developing CRC or different related cancer types (19). In the present study, a CRC-affected individual from a fulfilled Amsterdam II criteria pedigree, showing Lynch-associated MSI and IHC phenotypes, underwent whole-exome sequencing (WES). The analysis of WES data revealed no pathogenic variants in any of the MMR genes. Further evaluation unveiled a frameshift variant in *WRN* (*RECQL2;* HGNC ID: 12791) gene as a potential candidate gene deserving subsequent considerations.

*WRN* encodes a DNA helicase enzyme from the highly conserved *RECQ* gene family and has a pivotal role in DNA repair and maintenance of genomic stability (29, 30). The *WRN* has both helicase and exonuclease activity and has been considered a genomic caretaker (31). Homozygous truncating variants in the *WRN* gene cause Werner syndrome (WS; OMIM 277700) manifests, which are characterized by early onset age-associated diseases such as cancer (32, 33). In addition to the elevated risk of developing cancer in WS patients, several studies have indicated a tumor suppressor-like function for *WRN*, while making it a prominent cancer-predisposing gene (34, 35). More importantly, various evaluations have suggested a crucial role for *WRN* associated with different types of familial cancers, specifically breast and ovarian cancers (36–38). However, the association of germline *WRN* gene variants with familial CRC has not been established well. Herein, we aimed to introduce a truncating variant in *WRN* (NM_000553:exon9:p.R389Efs*3) as a pathogenic candidate based on ACMG standard guideline (39) in a patient affected by early onset familial CRC.

## Subject and Methods

### Ethics Statement

The Ethics Committee of Shahid Chamran University of Ahvaz approved the present study (Ref. EE/97.24.3.70385/scu.ac.ir). After genetic counseling and obtaining a comprehensive family history, we received informed written consent.

### Clinical Evaluation

A CRC affected patient in a family with different types of Lynch-associated cancer (n = 6), CRC, breast, and brain, was recruited from our other study on MSI optimizing in Lynch suspected patients (not published yet). An expert clinical pathologist assessed clinical and pathological data of surgically resected tumors.

### DNA Extraction and MSI Analysis

Whole blood and the formalin-fixed-paraffin-embedded (FFPE) blocks were obtained, and DNA extraction was performed based on standard methods. The evaluation of the quality and quantity of the extracted DNA was performed by agarose gel electrophoresis and nanodrop (Thermo Scientific, NanoDrop One C). Promega MSI Analysis version 1.2, (Promega, USA) was utilized containing fluorescently labeled five mononucleotide and two pentanucleotide markers to compare MSI status on tumoral and matched normal tissues according to the protocol. Fragment analysis was performed using the ABI3500 genetic analyzer (Applied Biosystems) and gene marker software V 1.85. The cutoff of MSI-H classification was two or more unstable markers (≥30%) (40).

### Immunohistochemistry

Immunohistochemistry analysis was carried out to evaluate MMR protein expression employing mouse monoclonal antibodies against MSH2 (Leica Biosystems: Novocastra, UK, Lyophilized, Product Code (PC): NCL-MSH2; at 1/80 dilution), MLH1 (Leica Biosystems: Novocastra, UK, Liquid, PC: NCL-L-MLH1; at 1/100 dilution), MSH6 (Leica Biosystems: Novocastra, UK, Liquid, PC: NCL-L-MSH6; at 1/100 dilution), and PMS2 (Leica Biosystems: Novocastra, UK, Liquid, PC: NCL-L-PMS2; at 1/100 dilution). In brief, 4 µm-thick FFPE sections were initially de-waxed and rehydrated by using xylene and graded ethanol, respectively. The sections were placed in citrate buffer (10 mmol/L, pH 6) and preheated at 99°C for 30 min. Endogenous peroxidase activity was quenched by exposure to 3% hydrogen peroxidase for 5 min. Subsequently, the sections were incubated for 20 min at room temperature with monoclonal antibodies. The detection was performed by applying Ultra-vision streptavidin-biotin peroxidase kit (Dako, Carpinteria, CA) according to the manufacturer’s instructions. The complete absence of each MMR protein expression in neoplastic cells was considered MMR deficient (7).

### Whole Exome Sequencing and Data Analysis

The DNA extracted from peripheral blood was fragmented with the hydrodynamic shearing System (Covaris, Massachusetts, USA). Afterward, Agilent SureSelect Human All Exon kit (Agilent Technologies, CA, USA) was used for enriching the exonic library. The uncaptured DNA was washed, and the trapped DNA was subjected to sequencing on Illumina NovaSeq 6000 with over 100× coverage. The generated fastq file was mapped to the reference genome (hg19, NCBI Build 37) employing the BWA aligner tool. The identified single nucleotide (SNV) and Indel variants, detected by GATK- v3.1, were annotated by ANNOVAR.

Primarily, based on the ACMG guidelines for genetic testing of inherited CRC (41), and the ACMG standard and guidelines for the interpretation of sequence variants (39) we evaluated the pathogenicity of the identified variants in highly associated genes, *MSH2*, *MSH6*, *EpCAM*, *MLH1*, and *PMS2*. We also searched the InSIGHT database for the known MMR gene pathogenic variants. Furthermore, the coverage of sequencing in the mentioned gene regions was assessed using IGVD software. Afterward, public databases were employed, including dbSNP version 147, 1000 Genome Project phase 3, and Exome Aggregation Consortium (ExAC), to filter the variants based on minor allele frequency (MAF < 1%). Further filtration was performed based on the functional consequence and inheritance pattern. The Human Gene Card Database (GeneCards) was utilized to prioritize the remaining gene variants according to the clinical phenotype. Catalog of Somatic Mutation Database (COSMIC), Human Gene Mutation Database (HGMD), and ClinVar were investigated, and literature was exhaustively reviewed to figure out deleterious variants associated with the disease.

### MLPA

Multiplex ligation-dependent amplification assay (MLPA) was performed using *MLH1/MSH2* MLPA probe mix (MRC-Holland Amsterdam, the Netherlands, Lot N: D1-0815). According to the manufacturer’s instruction, the thermal cycles were performed at 98°C to denature 5 µl of genomic DNA and prepare it for hybridization at 60°C with MLPA probes. Following overnight hybridization, the ligation process was done at 54°C for 15 min. After the inactivation of ligase enzyme at 98°C Polymerase mastermix and SALSA PCR primer mix were added, followed by 35 cycles of thermocycler program; including 30 s at 95°C; 30 s at 60°C; 30 s at 72°C; and 20 min at 72°C. PCR products were separated *via* capillary electrophoresis on ABI 3500 genetic analyzer (Applied Biosystems, USA). The data were collected and analyzed through Coffalyser software.

### Sanger Sequencing

Primer 3 software was used to design appropriate primers for *WRN *exon 9 forward (F-AAAAGCTTCACAGTTTGTCCTTG) and reverse (R-GGGAATAAAGTCTGCCAGAACC) primers. To design the relevant forward and reverse primers of *MSH2* (F-TGGGACATAGCAGGCCATAT and R-GCGGCAACCAATCATAAGCA) and *MSH6* (F-CTGGGTCAAAGGGCTCAGAT and R-TCGGACGGAGCTCCTAAAAG) gene promoter regions (42–44) Primer 3 software was used. PCR reaction was carried out using specific primers. The PCR products were subjected to Sanger sequencing on an ABI3500 genetic analyzer (Applied Biosystems, USA) instrument according to the standard protocols. Obtained sequences were aligned against the human reference genome (hg19, NCBI Build 37) using MEGA-X software.

## Results

### Clinical Features and Molecular Profiling

**Figure 1:**
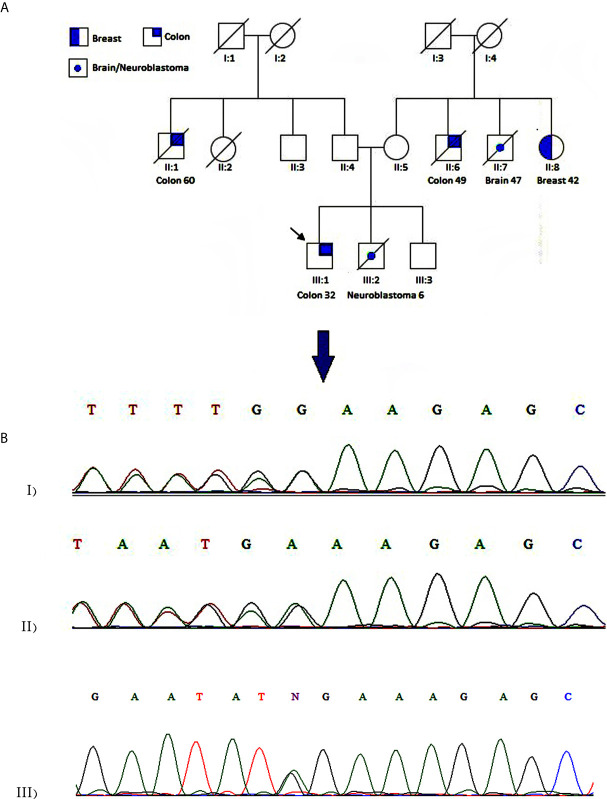
Case presentation **(A)** Pedigree of the family. The panel shows a family affected with different types of cancer. An arrow designates the studied patient. Colon and breast cancers are indicated by half and quarter filled colors, respectively. A central spot demonstrates neuroblastoma or brain cancer. **(B)** Chromatogram showing heterozygous c.1163delA mutation in WRN gene for proband (I) and patient II:8 (II). A homozygous wild-type variant was detected in the proband’s unaffected brother (III).

The patient was a 32-year-old man from an inbreed family, subjected to partial colectomy due to CRC adenocarcinoma. Amsterdam II criteria were fulfilled considering maternal and paternal family history (**Figure 1A**). CRC was diagnosed at the age of 32 for the proband (III:1). The affected individuals existed in two generations; two of them were first-degree relatives. The proband’s younger brother (III:2) died because of neuroblastoma at the age of 6. Six individuals were affected with different types of cancers in this family, and five of whom were diagnosed with malignant lesions under the age of 50. The spectrum of extracolonic cancers included neuroblastoma, breast, and brain tumors in this family. A 42-year-old female was diagnosed with breast carcinoma at the age of 38 (II:8). Two individuals, II:6 and II:1, passed away because of CRC at the age of 60 and 49 years, respectively. II:7 died due to brain cancer at the age of 47.

**Figure 2:**
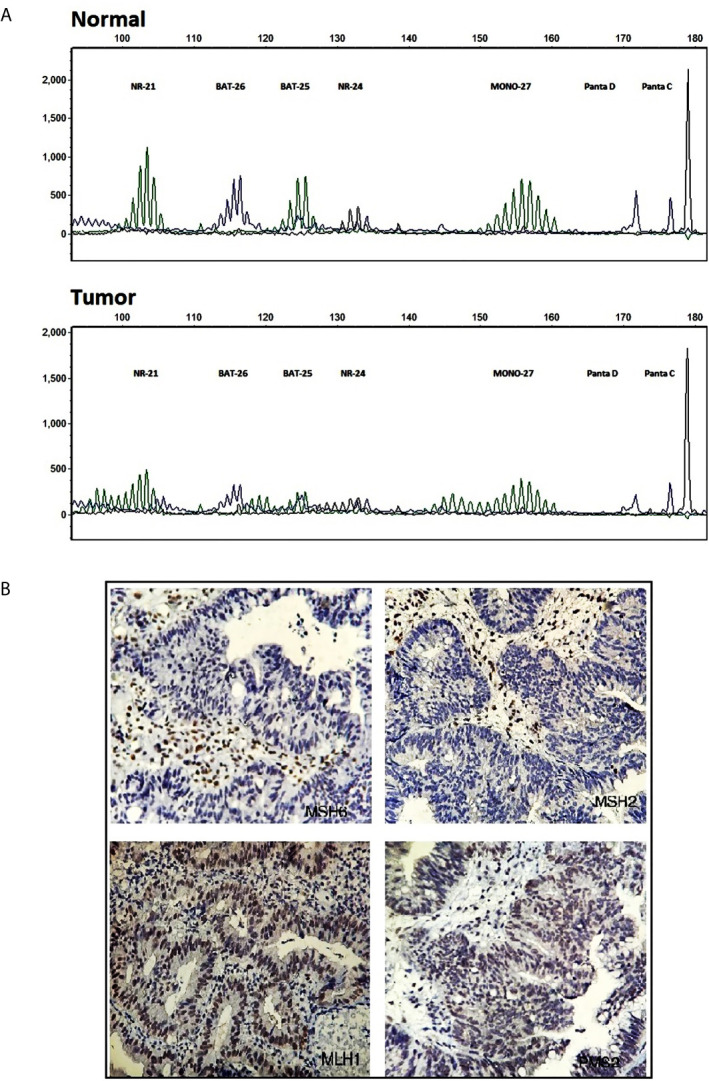
Molecular testing results. **(A)** Fragment profile of the Promega MSI analysis system (GeneMarker v1.85 Software). Instability is detectable at tumoral tissue (bottom) compared to normal at BAT-25, BAT-26, NR-21, NR-24, and MONO27 markers. **(B)** Immunohistochemistry staining for MMR system proteins, MSH2, MSH6 (mutant) MLH1, PMS2 (intact), in tumoral tissue.

MSI analysis was performed on the DNA samples extracted from FFPE blocks of the proband (III:1). The result implied that instability existed in five Promega mononucleotide markers, BAT-25, BAT-26, NR-21, NR-24, and MONO-27, in the tumoral DNA compared to the normal tissue (**Figure 2A**) indicating MSI-H profile. IHC analysis using MLH1, MSH2, MSH6, and PMS2 antibodies showed loss of MSH2 and MSH6 proteins in CRC tumor tissue. MLH1 and PMS2 antibodies represented the intact expression of these two proteins (**Figure 2B**).

### MMR Evaluation

**Figure 3:**
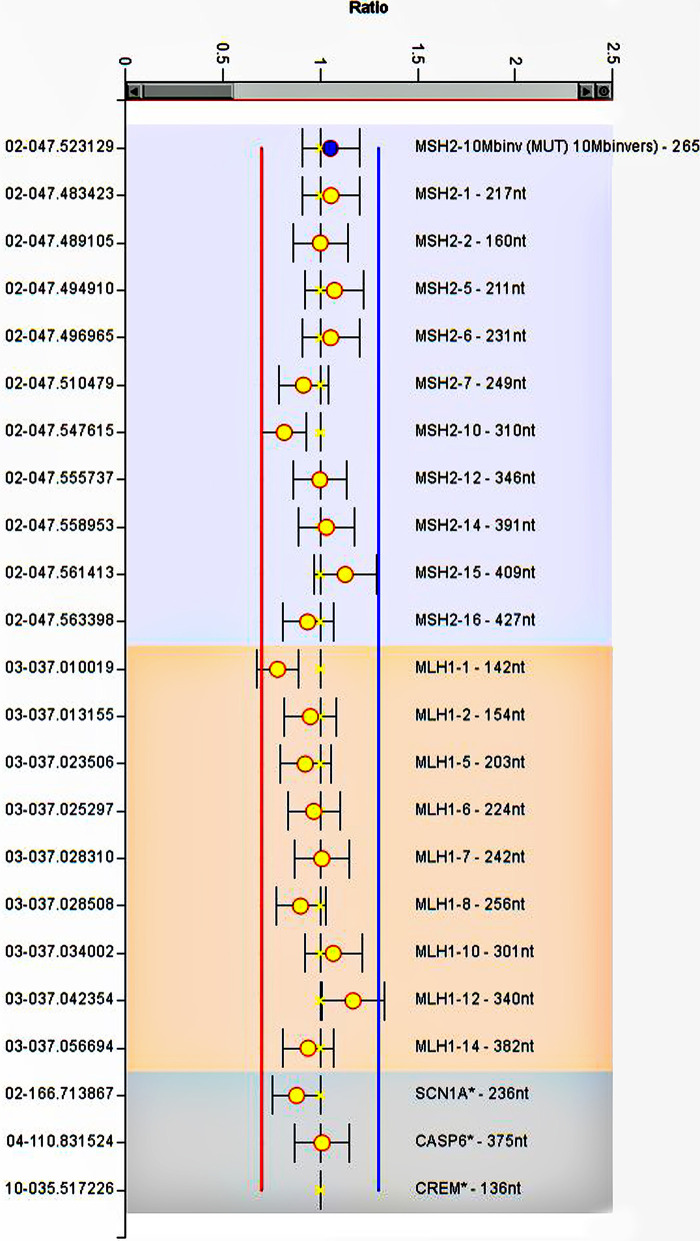
MLPA analysis of *MLH1* and *MSH2*. The image represents no deletion or duplication at gene regions. The error bars show standard deviation. Ratios of the signals normalized to the control reference individual.

The CRC-affected patient (III:1), whose clinical and molecular examination suggested hereditary Lynch phenotype, was subjected to WES. Considering full coverage Lynch-association gene loci, we achieved 99 variants, eight of which were in the exonic regions. Among these MMR exonic variants, there were no deleterious variants except for three non-synonymous variants in *MLH1*, *MSH6*, and *EpCAM* with MAF >1%. Furthermore, we performed Sanger sequencing by using specific primers of the promoter regions of *MSH2* and *MSH6* genes (44, 45). Sequence alignment against the human reference genome (hg19, NCBI Build 37) detected no deleterious variant in the corresponding regions (**Supplementary Figure 1**). In general, none of these variants were recognized as pathogenic based on the ACMG guideline and the InSIGHT database (39). To trace down large deletion or duplication MLPA was performed on the patient’s genomic DNA, and no deleterious variants were detected at *MLH1* or *MSH2* genes (**Figure 3**).

### Pathogenic Variant Identification and Validation

Screening for prioritized genes provided us a list of candidate exonic variants. Regardless of synonymous and non-synonymous variants, we narrowed down our investigation by incorporating genes harboring truncating variants, including nonsense, start loss, splice site, or frameshift variants (**Table 1**). After a comprehensive database survey and comprehensive literature review, we reached a frameshift variant in *WRN* gene exon9:c.1163delA (p.R389Efs*3). It introduced a truncating gene product supported by functional studies to render pathogenicity (34, 35, 39, 46). Population data indicates a frequency lower than 0.0001 in genomeAD-Exome Asian and ExAC Asian databases. Moreover, no frequency was reported in genomeAD-genome East Asian and in-house databases Iranome for instance. The variant was represented as pathogenic or likely-pathogenic at the ClinVar database (Accession number: RCV000232269.6) for WS. Sanger sequencing data using designed primers confirmed exon9:c.1163delA (GRCh37.p13) as diagnosed through WES in a heterozygous pattern in the proband and patient II:8 (**Figure 1B**). Proband’s unaffected brother (III:3), aged 38 was homozygote for the wild type of the variant. Unfortunately, we could not have access to the DNA samples of other family members to complete the cosegregation analysis.

**Table 1:** Truncating heterozygous variants (i.e., putative frameshifts, stopgain, nonsense mutations) in candidate CRC predisposing genes.

Gene	Accession number	Chr	Ref	Var	Mutation position	Mutation type	Protein effects	Hom/Het	dbSNP	MAF	ACMG guideline interpretation
MEGF6	NM_001409	1	G	A	3413648	Stopgain	p.Q1173*	Het	–	–	VUS
TTN	NM_001267550	2	C	G	179528741	Splice Site	c.36364+1G>C	Het	rs777040601	0.000045	VUS
AKAP9	NM_005751	7	C	_	91691592	Frameshift	p.I1924Lfs*20	Het	–	–	VUS
WRN^a^	NM_000553	8	A	–	30938706	Frameshift	p.R389Efs*3	Het	rs558267186	0.0000479	Pathogenic
SDHD	NM_001276504	11	T	A	111965692	Stoploss	p.X160R	Het	–	–	VUS

Chr, chromosome; Ref, reference allele; Var, variant allele; Hom/Het, homozygous/heterozygous MAF; minor allele frequency; VUS, variants of uncertain significant.

a WRN gene variant were validated with Sanger sequencing.

## Discussion

While many cancers have a positive family history, pathogenic variants in the well-known cancer-predisposition genes are responsible for up to 10% of hereditary cancers. The number of genes involved in cancer susceptibility is growing and further clinical and molecular evidence is needed to explain the physiopathology of these variants in cancer (47).

In the present study, we investigated the genetic etiology of the familial CRC in a pedigree meeting the Amsterdam criteria (48, 49). Since the clinical and pathological data and molecular analysis strongly implicated the LS (41), we originally focused on the evaluation of MMR system gene variants. Furthermore, we performed Sanger sequencing to assess the presence of any candidate pathogenic variant in the *MSH2* and *MSH6* gene promoters (43, 45). According to the InSIGHT database and the ACMG guideline, no pathogenic variant was identified in the exonic and regulatory elements of MMR genes. Moreover, MLPA testing was done. The data ruled out the presence of deleterious duplication or deletion in *MLH1* or *MSH2* genes (50). Accordingly, reanalysis of exome data through GeneCard and COSMIC dataset resulted in a list of relevant gene variants (**Table 1**). The subsequent assessment suggested that a *WRN* frameshift pathogenic variant (p.R389Efs*3) could be related to the etiology. Interestingly, the proband’s maternal aunt, II:8, suffering from breast cancer, was found to carry this variant.

Although *WRN* has been introduced as a candidate gene for hereditary breast cancer (37), the inheritance of the variant from the maternal side has sparked a debate on its role in developing colorectal cancer. The observation of dMMR phenotype might suggest the possibility of developing sporadic CRC due to bi-allelic somatic inactivation of *MSH2* and *MSH6* genes (12, 24). However, the positive history of cancer in this family and the patient’s young age weaken this assumption in the proband. Zimmer et al. showed that 56% of CRC tumors with bi-allelic mutant *WRN* gene presented MSI-H/dMMR status (51). The wide spectrum of reported cancers related to different tumor suppressor genes (*BRCA1*, *BRCA2*, *MSH2*, *MLH1*, etc.) would support the hypothesis that *WRN*, which has been previously confirmed as a candidate gene for hereditary breast cancer, may also lead to CRC (52, 53).

However, there remains a slight chance for the inheritance of an MMR gene pathogenic variant from the paternal side possibly not detected through the applied methods in the present study. According to several studies, MSI has a multi-pathway mechanism, and non-mutational events and implications of novel genes may explain MSI/dMMR tumors with no detectable germline mutation (32, 54, 55). For example, it was demonstrated that *BCL2* gene overexpression, commonly expressed in various tumor types, can downregulate the *MSH2* gene and subsequently reduce MMR protein cellular activity *via* the hypophosphorylation of pRb protein (56). Likewise, it was revealed that *WRN* gene silencing is linked to the MSI in CRC tumors and *in vitro* MSI models (54, 57). In this regard*, WRN* plays an essential role in the survival of MSI/dMMR cancer cell lines (not in MMR-proficient and MSS cells). It has been proposed that *WRN* is involved in an *EXO1* independent pathway for mismatch repair in MSI-H cells (58, 59). Besides, MSI-H/dMMR high frequency in the *WRN* mutant CRC tumors would support this hypothesis that *WRN* may be involved in the MSI events as an alternative pathway in undefined dMMR CRCs (51). Our finding in the present study will put forth *WRN* as a novel gene possibly leading to CRC associated with MSI, although its exact mechanism remains unknown.

The identified variant was interpreted as pathogenic based on the ACMG guideline. Since this premature stop codon variant disrupted the helicase domain, the protein function is lost (PVS1) (39, 60). The frequency of this variant was notably low in genomeAD-Exome Asian and ExAC Asian databases and even absent in genomeAD-genome East Asian population data (PM2). This variant was represented in ClinVar (Variation ID: 238117) associated with WS (PS3). The functional assessments indicated that the damaging effects on *WRN* gene products were attributed to genomic instability in colorectal tumor cells (35). Furthermore, aberrant *WRN* function due to hemizygous gene loss was demonstrated in advanced clinical CRC (61). In addition, the genetic instability revealed in cell lines from WS patients indicates haploinsufficiency for the *WRN* gene (32).

The defective *WRN*, a member of the *RecQ* helicase family, implicates WS (29). RecQ helicases are known as genome caretakers and play a critical role in genomic stability (62). The involvement of *WRN*, *BLM*, and *RecQL4* family members has been determined in the pathogenesis of well-defined genetic disorders with an elevated risk of cancer (63). The *WRN* gene is located at 8p12 and contains 35 coding exons. The protein comprises ATPase, RQC, and HRDC domains, as well as an N-terminal exonuclease domain. It was shown that *WRN* participates in several DNA metabolic pathways, including replication, recombination, and repair to preserve the genome (30, 31, 64). To date, 140 pathogenic and 31 likely pathogenic variants have been identified in the *WRN* gene based on the ClinVar database. These variants include deletion, duplication, indel, insertion, and single nucleotide substitution leading to the destabilizing or protein failure to localization to the nucleus. The recessive truncating variants are described in association with WS well, suggesting that complete loss of *WRN* is necessary to develop the disease (32).

It was determined that truncating variants, which destroy the helicase activity of *WRN, *participate in WS pathogenesis (32, 60). The complete loss of *WRN* in WS patients born with a germline homozygous deleterious variant will lead to premature senescence and neoplastic alteration (65). *WRN* inactivation can induce DNA double-strand breaks and subsequently activate DNA damage responses such as apoptosis and cell cycle arrest (57). WS patients are susceptible to a variety of cancer types, including colorectal, ovarian, pancreatic, skin, and breast cancers, as well as soft tissue sarcomas, osteosarcomas, and leukemia (65, 66). A common pathogenic variant was shown to play a pivotal role in the genesis of thyroid carcinoma in Japanese WS patients. Furthermore, the association of the follicular and papillary carcinomas with the *WRN* c.3139-1G>C (exon 26 skip) and c.1105C>T (p.R369*) variants was found in WS patients, respectively (67, 68). Considering the significant frequency of different neoplasms in WS patients, WS was considered a heritable cancer predisposition syndrome. However, limited studies have investigated the role of *WRN* in developing cancer in non-WS individuals. Experiments revealed a degree of genetic instability for carriers of the *WRN* heterozygous pathogenic variants. These studies demonstrated that *WRN* inactivation promotes genetic instability in different human somatic cell lineages (32, 54, 68). However, it should be noted that *WRN* has a tumor suppressor function, and the loss of its second wild-type allele is necessary for carcinogenesis events to fulfill Knudson’s two-hit hypothesis of tumor suppressor genes (11).

Notably, epigenetic inactivation of the *WRN* gene was introduced as a common event in sporadic colorectal tumorigenesis as described for other DNA repair tumor suppressor genes, *hMLH1* and *BRCA1*, involved in hereditary cancers (34). Furthermore, the aberrant function of the *WRN* gene in MSI-H CRC tumors, as a result of frameshift variants, suggested the probability of *WRN* contribution at MSI-H tumorigenesis as a putative tumor suppressor (35). Additionally, *WRN’s* contribution to breast cancer susceptibility has been demonstrated by Ding et al. They reported that a *WRN* specific variant (rs9649886), situated in the C-terminal of WRN, significantly affected breast cancer risk (36). Likewise, Zins et al. examined the significance of a *WRN* non-synonymous variant (rs1346044) with an elevated risk of breast cancer. They indicated that the risk of breast cancer increased in homozygous individuals due to the reduced helicase activity (38). Even though the potential role of *WRN* in promoting breast cancer was undetermined, more recently, a novel germline frameshift variant (p.N1370Tfs*23) has been identified in a Chinese family. The truncated variant was confirmed in three affected individuals and an unaffected first-degree relative. The identified variant was interpreted as pathogenic according to the ACMG guidelines, thereby introducing *WRN* as a potential candidate gene for hereditary breast cancer (37, 39).

In conclusion, hereditary cancer syndromes have provided a remarkable venue in cancer investigation, particularly in diagnostic and therapeutic strategies. Furthermore, the identification of at-risk individuals through germline mutation testing is a key to success in the prevention, early detection, and management of patients (21, 26). However, *WRN *has not been addressed as a hereditary CRC predisposing gene so far. We noticed a *WRN* pathogenic variant as a disease-causing variant in a familial CRC patient. Our data could be further authenticated by following up the family, performing additional functional studies, and precisely detecting the loss of heterozygosity feature in colorectal tumor cells. Nevertheless, the potential role of *WRN* in CRCs remains elucidated, and further studies are needed to reveal its role in hereditary cancers, HNPCC subtypes in particular.

## Data Availability Statement

The original contributions presented in the study are included in the article/**Supplementary Material**; further inquiries can be directed to the corresponding author/s.

## Ethics Statement

The study involving human participants was reviewed and approved by the Ethics Committee of Shahid Chamran University of Ahvaz (Ref. EE/97.24.3.70385/scu.ac.ir). The patients/participants provided their written informed consent to participate in this study.

## Author Contributions

MS and MT conceived and designed the study, obtained funding, developed methodology, reviewed, and revised the manuscript. MZ and ME managed patients and provided facilities. MN drafted the manuscript. HG revised the manuscript. MN, ZA, and PM performed molecular techniques and interpretation of data. All authors contributed to the article and approved the submitted version.

## Funding

Shahid Chamran University of Ahvaz (grant number 97/3/02/26247) and Isfahan University of Medical Science (grant number 195216) financially supported this work.

## Conflict of Interest

The authors declare that the research was conducted in the absence of any commercial or financial relationships that could be construed as a potential conflict of interest.
