# Supplementary material for: WRN Germline Mutation Is the Likely Inherited Etiology of Various Cancer Types in One Iranian Family
Source: Front Oncol. 2021 Jun 7;11:648649. doi: 10.3389/fonc.2021.648649 (PMC8215443; doi:10.3389/fonc.2021.648649)
Supplement: Supplementary file 1 [file DataSheet_1.docx]

**Supplementary Figure 1.** Sanger sequencing results of *MSH2* (A) and *MSH6* (B) gene promoters were compared to RefSeq (hg19, NCBI Build 37) using multiple sequence alignments.

**A**

Sangerseq-F 1 GTAGAATAAACCCGTAATCCCGATGTTGGCAGTTTGCTTAGAAAGAAAAAGGGAGGCAGT
SangerSeq-R 1 GTAGAATAAACCCGTAATNCCGATGTTGGCAGTTTGNTTAGAAAGAAAAAGGGAGGCAGT
RefSeq 1 GTAGAATAAACCCGTAATCCCGATGTTGGCAGTTTGCTTAGAAAGAAAAAGGGAGGCAGT


Sangerseq-F 61 CGGAGAGGGGCACACGTTTTAACAAAATACTGGGAGGAGGAGGAAGGCTAGTTTTTTTTT
SangerSeq-R 61 NGGAGAGGGGCNCNCGTTTTAACAAAATNCTGGGAGGAGGAGGAAGGNTAGTTTTTTTTT
RefSeq 61 CGGAGAGGGGCACACGTTTTAACAAAATACTGGGAGGAGGAGGAAGGCTAGTTTTTTTTT


Sangerseq-F 121 NGTTTTCAAGTTNCCTTCTGATGTTACTCCCATGCTTCCGGGCACNTTACNAGCTCANNG
SangerSeq-R 121 TGTTTTCAAGTTTCCTTCTGATGTTACTCCCATGCTTCCGGGCACATTACGAGCTCAGTG
RefSeq 121 TGTTTTCAAGTTTCCTTCTGATGTTACTCCCATGCTTCCGGGCACATTACGAGCTCAGTG


Sangerseq-F 181 CCTGCCGNAAATCTCCCACCTGGNGGCAACCTACCCTTGCATACNCCCCACCCAGGGGCT
SangerSeq-R 181 CCTGCCGGAAATCTCCCACCTGGTGGCAACCTACCCTTGCATACACCCCACCCAGGGGCT
RefSeq 181 CCTGCCGGAAATCTCCCACCTGGTGGCAACCTACCCTTGCATACACCCCACCCAGGGGCT


Sangerseq-F 241 TCAAGCCTTGCANCTNANTAAACACANAAAGGANCTCTACTAAGGATGCNCGNCNGCGGG
SangerSeq-R 241 TCAAGCCTTGCAGCTGAGTAAACACAGAAAGGAGCTCTACTAAGGATGCGCGTCTGCGGG
RefSeq 241 TCAAGCCTTGCAGCTGAGTAAACACAGAAAGGAGCTCTACTAAGGATGCGCGTCTGCGGG


Sangerseq-F 301 TTTCCNCGCGACCTAGGCNCAGGCNTGCNCA
SangerSeq-R 301 TTTCCGCGCGACCTAGGCGCAGGCATGCGCA
RefSeq 301 TTTCCGCGCGACCTAGGCGCAGGCATGCGCA

**B**

SangerSeqF 1 AAAACTTTGTACAGCTGCAGGCTTTCACCATACACAACAGCATCGCTAACGAATGCTATT
SangerSeqR 1 AAAACTTTGTACAGCTGCAGGCTTTCACCATACACANCAGCATNGNTAACGAATGNTATT
RefSeq 1 AAAACTTTGTACAGCTGCAGGCTTTCACCATACACAACAGCATCGCTAACGAATGCTATT


SangerSeqF 61 ACAATATTCATTTAGCGTTTACCAAGTGCCTACTCTATACAAATCTTGAGAATACAACGT
SangerSeqR 61 ACAATATTCATTTAGCGTTTACCAAGTGCCTNCTCTATNCAAATCTTGAGAATACAACGT
RefSeq 61 ACAATATTCATTTAGCGTTTACCAAGTGCCTACTCTATACAAATCTTGAGAATACAACGT


SangerSeqF 121 GAAGGTGAACTGCTGACTAAAGTTTGGTCCCTTTCGCTCCGTCTCCTTGCGAAAATGCTC
SangerSeqR 121 GAAGGTGAACTGCTGACTAAAGTTTGGTCCCTTTCGCTCCGTCTCCTTGCGAAAATGCTC
RefSeq 121 GAAGGTGAACTGCTGACTAAAGTTTGGTCCCTTTCGCTCCGTCTCCTTGCGAAAATGCTC


SangerSeqF 181 TAACGGCAGGAGGTCACGCGAGCGCTGGACGCGTTTCTCCCCGCGAGCCCCTTTCCGAGG
SangerSeqR 181 TAACGGCAGGAGGTCACNCGAGCGCTGGACGCGTTTCTCCCCGCGAGCCCCTTTCCGAGG
RefSeq 181 TAACGGCAGGAGGTCACGCGAGCGCTGGACGCGTTTCTCCCCGCGAGCCCCTTTCCGAGG


SangerSeqF 241 CCTTTCGGGTCCCCCCGGTTATCCCCGCCCGGGCGGTGCGCGCCCCCGCTGTTCCCGCTT
SangerSeqR 241 CCNTTCGGGTCCCCCCGGTTATCNCCGCCCGGGCKGTGCGCGCCCCCGCTGTTCCCGCTT
RefSeq 241 CCTTTCGGGTCCCCCCGGTTATCCCCGCCCGGGCGGTGCGCGCCCCCGCTGTTCCCGCTT


SangerSeqF 301 CCGCTCCAGANAGGCAGGGCTTTCCGAGCNTGNTAGCCCCGCGGCCGCAACTAACCCCGG
SangerSeqR 301 CCGCTCCAGAGAGGCAGGGCTTTCCGAGCCTGCTAGCCCCGCGGCCGCAACTAACCCCGG
RefSeq 301 CCGCTCCAGAGAGGCAGGGCTTTCCGAGCCTGCTAGCCCCGCGGCCGCAACTAACCCCGG


SangerSeqF 361 GTCGGAGTGTTCCGGCCCGGCCAGCCCCGCGGCGTGAGGGAAGGGGAGCTCAGCAGTTCC
SangerSeqR 361 GTCGGAGTGTTCCGGCCCGGCCAGCCCCGCGGCGTGAGGGAAGGGGAGCTCAGCAGTTCC
RefSeq 361 GTCGGAGTGTTCCGGCCCGGCCAGCCCCGCGGCGTGAGGGAAGGGGAGCTCAGCAGTTCC


SangerSeqF 421 CCGCGCGGGGCCCAGGCGTCGGCGGCAGGGCGGGCCCCTCACCGCCAGCGTGCCAGCCCC
SangerSeqR 421 CCGCGCGGGGCCCAGGNGTCGGCGGCAGGGCGGGCCCCTCACCGCCAGCGTGCCAGCCCC
RefSeq 421 CCGCGCGGGGCCCAGGCGTCGGCGGCAGGGCGGGCCCCTCACCGCCAGCGTGCCAGCCCC


SangerSeqF 481 GCCCCTACCCACCAGNGNGCCAGCCCCGCCCTTCCCCACGTCGCCGCGCGCCCGGGGGCG
SangerSeqR 481 GCCCCTACCCACCAGTGTGCCAGCCCCGCCCTTCCCCACGTCGCCGCGCGCCCGGGGGCG
RefSeq 481 GCCCCTACCCACCAGTGTGCCAGCCCCGCCCTTCCCCACGTCGCCGCGCGCCCGGGGGCG


SangerSeqF 541 GGGCCTGGCGCGCACCGCCCGCGCACGGCGAGGCGCCTGTTGATTGGCCACTGGGGCCCG
SangerSeqR 541 GGGCCTGGCGCGCACCGCCCGCGCACGGCGAGGCGCCTGT--------------------
RefSeq 541 GGGCCTGGCGCGCACCGCCCGCGCACGGCGAGGCGCCTGTTGATTGGCCACTGGGGCCCG


SangerSeqF 601 GGTTCCTCCGGCGGAGCGCGC
SangerSeqR 581 -------------------TG
RefSeq 601 GGTTCCTCCGGCGGAGCGCGC
